# Does Time Heal Trauma? 18 Month Follow-Up Study of Syrian Refugees’ Mental Health in Iraq’s Kurdistan Region

**DOI:** 10.3390/ijerph192214910

**Published:** 2022-11-12

**Authors:** Harem Nareeman Mahmood, Hawkar Ibrahim, Azad Ali Ismail, Frank Neuner

**Affiliations:** 1 Department of Psychology, Clinical Psychology and Psychotherapy, Bielefeld University, 33501 Bielefeld, Germany; 2 Department of Clinical Psychology, Faculty of Science and Health, Koya University, Koy Sanjaq 46017, Iraq; 3 vivo International, 78430 Konstanz, Germany

**Keywords:** mental health, time, post-migration condition, refugee

## Abstract

The findings of longitudinal studies on traumatized refugees have shown that factors related to premigration, migration, and post-migration experiences determine changes in mental health over time. The primary aim of this follow-up study was to examine the potential change in the prevalence rates of probable PTSD and depression among Syrian refugees in Iraq. An unselected group of N = 92 Syrian adult refugees was recruited from Arbat camps in Sulaymaniyah Governorate in Iraq’s Kurdistan Region, and then interviewed at two different time points between July 2017 and January 2019. Locally validated instruments were used to assess traumatic events and mental health symptoms. The primary results showed no significant change in the mean scores of PTSD and depression symptoms from the first measurement to the second measurement over the course of 18 months. On the individual level, no reliable change was found for either PTSD or depression symptoms in more than three-quarters of the participants (78.3% and 77.2%, respectively). New adversities and traumatic events that occurred over the 18 months between the interviews were a significant predictor of increasing trauma-related symptoms. After the flight from conflict settings, trauma-related disorders seem to be chronic for the majority of Syrian refugees. Further longitudinal studies are needed in order to identify specific risk factors that lead to maintaining or worsening mental health symptoms over time, and to explore effective therapeutic intervention methods for this traumatized population.

## 1. Introduction

Over a decade of Syria’s civil war and ongoing conflicts have forced more than 13.4 million citizens to leave their homes, half of whom fled from their country [1]. Iraq has served as a country of reception for 263,783 Syrian refugees, almost all of them residing in the Kurdistan Region of Iraq (KRI) [2]. It has been well documented that refugee populations are at a greater risk of exposure to various traumatic events before migration, during the flight, and after resettlement in the host country [3,4,5,6,7]. This increased risk contributes to the fact that refugees are more vulnerable to developing mental health problems than the general population [8]. Findings from several meta-analyses and systematic reviews conducted in different countries have concluded that refugee populations suffer from mental disorders up to ten times more often than the general population [9,10].

Post-traumatic stress disorder (PTSD) and depression are the most prevalent and comorbid mental health problems in the aftermath of a traumatic event, particularly among war-affected populations, including refugees [11,12]. A systematic review and meta-analysis of refugees and asylum seekers identified high overall prevalence rates of PTSD (31.5%) and depression (31.5%) [6]. Since Syrian refugees have gone through bloody conflict, high levels of mental health problems are expected [13,14]. Indeed, recent evidence has confirmed the high prevalence of mental distress among Syrian refugees [4,5,13,15,16,17]. Based on a systematic review of adult Syrian refugees who resettled in 10 countries, the overall prevalence rate of PTSD was 43%, and 40.9% for depression [18].

The trajectories of mental disorders are heterogeneous across longitudinal studies investigating refugees. While some studies reported a remission of mental health symptoms among refugees in the host countries [19,20,21,22], many studies found no significant change in mean symptoms [23,24,25,26,27,28], and some studies reported a worsening of mental health symptoms in refugee populations over time [29,30,31]. It is important to note that the analysis of the change in the mean scores of symptoms is not sufficient to determine the extent of change that occurs between two timepoints, since the improvement of some individuals might be balanced out by worsening in others, and thus appear as no change in the overall population. In fact, a longitudinal study by Kaltenbach and colleagues [32] among refugees in Germany has shown that, although the average severity of symptoms did not change over the period of one year, a statistically reliable change was found on the individual level, with an improvement of PTSD and depression documented for up to a quarter of participants, which was accompanied by a significant worsening in a similar number of refugees.

Beyond pre-migration traumatic events, cross-sectional studies have suggested that the post-migration conditions in the host country may be an important predictor of trauma-related symptoms among refugees [11,33,34,35,36]. In general, uncertain asylum status, living in shared refugee accommodations, lack of language skills, and integration issues are considered to be risk factors for high and persistent mental health disorders [37]. In addition, studies have indicated that experiencing additional trauma in the host country is a critical post-migration factor in worsening trauma-related symptoms in refugee populations [38,39]. Among Syrian refugees, factors such as racial discrimination, integration difficulties, language barriers, loss of social relationships, culture shock, fear of deportation, long stays in refugee camps, and high living costs contributed to the burden of psychological disorders [4,5,17,40,41].

However, the causality of these findings is unclear, and longitudinal studies of refugees are inconsistent in identifying post-migration predictors that cause a change in mental health trajectory. A recent study among refugees in Australia reported that post-resettlement PTSD and depression symptoms develop with an increase in the level of interpersonal post-migration difficulties [42]. Further, longitudinal studies identified that being female, daily stressors, difficulties in asylum procedures and social isolation were significant predictors of maintenance or worsening of trauma-related symptoms over time [26,28,43,44]. Other, longitudinal studies among traumatized refugees found that a new traumatic or stressful life event in the aftermath of initial trauma significantly increases pre-existing trauma-related symptoms [31,38,39,45]. Regarding Syrian refugees, a longitudinal study in Germany found that shorter duration of residence permission and higher perceived discrimination were among the most significant post-migration predictors of psychological stress over 1.5 years [23].

The main aim of the present study is to determine the change of mental disorders among Syrian refugees residing in the KRI, taking into account their previous traumatic experiences and potential adversities and traumatic events in camps. To the best of our knowledge, this is the first follow-up study screening the prevalence of mental disorders over time among Syrian refugees living in KRI camps. Although there are a few follow-up studies on psychiatric problems among Syrian refugees and other asylum seekers in Germany (e.g., [23,44]), the post-migration conditions in Iraqi camps are different. Since the host community in the KRI has already faced numerous political and economic crises, immigration of a large number of Syrian refugees added additional pressure on the local governorates’ authorities. Consequently, refugees have been hosted with limited livelihood resources [46,47]. This is in addition to the lack of sufficient psychotherapeutic services in the camps. Based on the high prevalence of mental disorders among Syrian refugees in Iraq (e.g., [5]), the absence of adequate intervention, and difficult living conditions in the camps, we hypothesized the persistence of mental disorders over time.

## 2. Methods

### 2.1. Participants and Procedures

The study participants were 92 Syrian refugees (46 married couples) recruited from Arbat camps in Sulaymaniyah Governorate in the KRI at two different time points (t1: July 2017 and t2: January 2019). The current study was a part of an extensive survey that was included in the first measurement, with a total of 988 Syrian refugees (494 married couples) who were recruited for the larger study. The present study uses a subsample of that larger body of participants. Detailed information about the sampling, the interviews, and the procedures are described elsewhere [5,48,49,50,51]. A random selection of N = 100 tents was chosen for the follow-up interview. All interviews followed the same procedure and protocol as the first measurement. Out of the 100 targeted participants, 92 Syrian refugees (46 couples) were available for the follow-up interview. The follow-up sample had not received any psychotherapy during the last 18 months. Approximately 85% of the participants did not have regular monthly income at both measurements. For detailed information about the sample characteristics, see Table 1.

### 2.2. Instruments

In addition to questions about socio-demographic variables such as gender, age, education, and migration background, the following instruments were used at both measurement times: The Kurdish Kurmanji and Arabic versions of the War and Adversity Exposure Checklist (WAEC) was employed in order to assess war-related and non-war-related traumatic events. The WAEC consists of 26 items (10 war-related and 16 non-war-related traumatic events) with two answer options (“Yes” and “No”), which were validated and developed by Ibrahim and colleagues for the displaced people of the Middle East [49]. Given that the participants were in camps far from warfare for the previous 18 months, only non-war traumatic events (16 items) during the past 18 months were analyzed in the second measurement. The checklist’s internal consistency among the current sample at each measurement time was 0.78 and 0.84, respectively.

The Kurdish Kurmanji and Arabic versions of the PTSD Checklist for DSM-5 (PCL-5) [50] were used to evaluate PTSD symptoms at both measurement points. The PCL-5 consists of twenty self-report items with a 5-point Likert scale for each item, ranging from ‘‘Not at all’’ (0) to ‘‘Extremely’’ (4). Ibrahim and his colleagues validated the checklist for the Middle Eastern population. Based on the calibration above, a cut-off score of 23 is the optimal fit for a diagnosis of PTSD among the Arab and Kurdish communities. High internal consistency (0.88 and 0.93, respectively) was obtained at both measurement times.

The depression subscale of the Kurdish Kurmanji and Arabic versions of the Hopkins Symptom Checklist-15 (D-HSCL-15) [50] were used to assess depression symptoms at both measurement times. The scale consisted of 15 items, and each item had four categories for the answer (“Not at all,” “A little,” “Quite a bit,” “Extremely”) rated from 1 to 4, respectively. The final score is determined by dividing the total of all item scores by the number of items (the final score ranges from 1.00 to 4.00) [52]. The original cut-off score of 1.75 (score of 26.25) is accounted for in diagnosing major depressive disorder clinically [53,54]. Although the instrument was not explicitly validated and standardized for our targeted population, high internal consistency (0.88 and 0.89, respectively) was achieved at both measurement times among the current sample.

### 2.3. Statistical Analysis

The statistical analysis was calculated using the SPSS program, version 28. Exploratory data analysis was performed to check the normality of the mental health scores. Descriptive analyses were carried out to profile the sociodemographic variables, number of experienced traumatic events, and mean severity of the sample’s mental health. The Wilcoxon signed-rank test was used to check the overall changes in mental health scores over time when the values were not normally distributed. The paired sample t-test was used for the same purpose when the values were normally distributed. The Reliable Change Index (RCI) was used to evaluate the change over time of an individual score [55]. Thus, the Standard Error of Difference (SED) formula was applied to determine the individual change in symptoms of PTSD and depression over time. Point-biserial correlation and Spearman-rank correlations were applied to check the possible associations between sociodemographic, traumatic events, and mental health variables. The change score variable for each PTSD and depression symptom was computed by calculating the second time score minus the first time (t2 − t1). After testing the assumptions, hierarchical multiple regression analyses were carried out to determine the main predictors in the change scores of PTSD and depression symptoms.

## 3. Results

### 3.1. Traumatic Experiences

At t1, out of 26 war-related and non-war-related traumatic events, participants were exposed to six traumatic events on average (M = 6.3, SD = 3.15, ranging from 0 to 13). Further, 97.8% of the participants reported at least one traumatic experience, and 86.9% reported being exposed to three or more traumatic events. The most frequent traumatic events were: ‘‘forced separation from first-degree relatives because of the war’’ (68.5%), ‘‘witnessing fire or explosion” (66.3%), ‘‘transportation accident’’ (62%), and ‘‘exposure to armed combat (e.g., bombing and airstrikes) during the war” (54.4%).

At t2, out of 16 non-war traumatic events in the last 18 months, participants reported more than one traumatic event on average (M = 1.05, SD = 1.18, ranging from 0–5). Specifically, 63% of the participants reported one traumatic experience, and 13% reported three or more traumatic experiences. The most common traumatic events were ‘‘experiencing a natural disaster ‘‘ (59.8%), ‘‘witnessing severe transportation accidents’’ (17.4%), and ‘‘experiencing the life-threatening illnesses of a loved one.’’ (15.2%).

### 3.2. Mental Health Symptoms over Time

At t1, the mean severity of PTSD symptoms in the present sample was 26.96, SD = 14.05 (range: 1–58). Using the validated cut-off score of 23 [50], 54.3% of the participants met DSM-5 symptom criteria for a probable diagnosis of PTSD. At t2, the mean severity of the PTSD symptoms was 24.22, SD = 16.23 (range: 0–62), and 53.3% of the participants met DSM-5 symptom criteria for a probable diagnosis of PTSD. The Wilcoxon signed-rank test results showed no significant change in the overall scores of PTSD symptoms *Z* = −1.29, *p* = 0.199 from the first measurement to the second measurement.

At t1, the mean severity of depression symptoms was 29.4, SD = 9.21 (range: 15–57). Using the instrument’s original cut-off score of 1.75 (score of 26.25), more than half of the participants (59.8%) met the criteria for a probable diagnosis of major depressive disorder. At t2, the mean severity of depression symptoms was 29.65, SD = 8.52 (range: 15–50), and 64.1% of them met or surpassed the cutoff score, indicating a probable major depressive disorder. The Wilcoxon signed-rank test showed no significant change in the overall depression symptoms scores: *Z* = −0.43, *p* = 0.666 from the first measurement to the second measurement.

On the individual level, based on the RCI criteria, 78.3% and 77.2% of participants did not meet the criteria for a reliable change between baseline and 18-month follow-up in PTSD and depression symptoms, respectively; see Table 2 for more details.

### 3.3. Predictors of Mental Health Change Scores

Hierarchical regression analyses were carried out to evaluate the prediction of PTSD and depression change score (t2 − t1) from age, gender, number of traumatic events in the baseline measurement, and number of non-war traumatic events over the 18 months. As presented in the Table 3, the results of the first block revealed the model not to be statistically significant for the PTSD change score (*F* (1,90) = 1.65, *p* = 0.202, R^2^ = 0.02), or for the depression change score (*F* (1,90) = 2.90, *p* = 0.092, R^2^ = 0.03). The results of the second block analysis revealed the model to be statistically significant for the PTSD change score, (*F* (3,87) = 6.69, *p* < 0.001, R^2^ = 0.20), and also for the depression change score (*F* (3,87) = 4.56, *p* = 0.005, R^2^ = 0.16). Overall, in the second model, approximately 20% and 16% of the variance in PTSD and depression change scores, respectively, can be accounted for by the included predictor variables. The significant predictor of changes in both PTSD and depression scores was the number of non-war traumatic events experienced during the last 18 months. Further analysis was conducted to determine specific non-war traumatic events that significantly correlated with PTSD and depression change scores. The results of the zero-order coefficient showed that experiencing the life-threatening illnesses of a loved one was among the most detrimental traumatic events that correlated to PTSD (*r*(90) = 0.46, *p* < 0.001), and depression change scores (*r*(90) = 0.30, *p* = 0.003); see Table 4 for more details.

## 4. Discussion

To the best of our knowledge, this is the first follow-up study that studied a potential change in Syrian refugees’ mental health in KRI camps. Over half of the participants met criteria for a probable diagnosis of PTSD and depression at both measurement times, and no significant change was found in the overall mean scores of either disorder over 18 months of follow-up. In our sample, the prevalence rate of probable PTSD and depression exceeded the previously reported rates of probable PTSD and depression among Syrian refugees living in Western countries [56]. The severity and chronicity of the symptoms were consistent with several findings that indicated that mental disorders could be highly persistent in war refugees, even after several years of resettlement [36].

Even years after the war, the mental disorders among Syrian refugees living in Iraq seemed to remain stable over time, with remission in some individuals, but also worsening in others. Mean levels of PTSD and depression remained almost identical within the 18 months between the two measurement points, and no reliable change was found among three-quarters of the sample from the first to the second measurement. The persistence of the symptoms was also in line with the findings of a longitudinal study among Syrian refugees in Germany [23], and with previous studies on the long-term effect of mental disorders among various refugee populations [25,26,27,28,57], although some studies have found a moderate improvement [21,22] or worsening [30,31] of symptoms in refugees over time. It appears that time alone does not have a significant healing effect in the case of most traumatized refugees [12] in general. Therefore, effective psychotherapy, such as Narrative Exposure Therapy (NET) [58], is highly recommended for this war-traumatized population. However, professional psychotherapeutic care in post-conflict countries, such as Iraq, cannot provide the necessary care for all displaced people with existing services [59]. This gap can be filled by increasing the human capacity in mental health treatment settings through training courses on effective psychotherapy (e.g., NET as a culturally sensitive trauma-focused therapy) for local psychologists and mental health care staff.

The living conditions in Iraqi camps seem to have a negative effect on symptoms. Consistent with several studies that emphasized the post-migration situation for the maintenance of psychological symptoms [11,33,34,35,36], we found that the refugees were exposed to a range of stressors, adversities, and new traumatic events that were associated with a worsening of symptoms. Iraq is an instable country that is confronted with ongoing conflict. In addition, Syrian refugees are living under difficult conditions even when compared to the host population. Consequently, many Syrian refugees in Iraq are looking for a chance to migrate to Western countries. A prospective cohort study suggested that Syrian refugees’ mental health outcomes improved when they moved from Lebanon to Norway for resettlement [21], indicating that safety and stability in the refugees’ life plays a role in their recovery.

The number of new non-war-related traumatic events over the last 18 months was the only predictor of changing scores in PTSD and depression among current Syrian refugees. This is in line with previous longitudinal studies, which found that mental disorder symptoms in the aftermath of primary trauma will worsen following the experience of a new traumatic or stressful life event or events (e.g., [39,45]). Researchers described this as re-traumatization that increases pre-existing trauma-related symptoms for a long period of time [60,61]. Further, the results showed that specific traumatic events, such as witnessing and experiencing a life-threatening illness or injury post-migration, were among the most common non-war-related traumatic events that burden the mental health of Syrian refugees over time. Exposure to trauma may not only be a factor that correlates with the high prevalence of mental disorders, but post-migration socio-economic conditions may also play a role in the persistence or worsening of mental disorders in refugees [36]. Thus, the results suggest that residing in refugee camps in Iraq, although providing security from war-related risks, still remains a risk factor for refugees’ mental health, and it seems to interact with the length of stay to worsen mental illness symptoms [23]. Further, life in camps, although not comfortable for many, is in some ways stable and predictable. Life stability and predictability might explain some of the changes in PTSD symptoms. It seems that daily life consistency might help the stabilization phase of post-trauma recovery. Another factor contributing to persistent psychological disorders and distress among refugees is that only a small number of refugees receive qualified mental health treatment. As indicated in a cross-sectional study in Turkey, due to stigma and a lack of accessible services, most Syrian refugees with mental illnesses did not seek therapy, but instead relied on the belief that symptoms would resolve over time [62].

On the individual level, a reliable improvement was observed for 16.3% and 14.1% of individuals with PTSD and depression symptoms, respectively. The figures for worsening symptoms were 5.4% for PTSD and 8.7% for depression. This unsystematic change in the symptoms was in line with findings of previous longitudinal studies on refugees residing in European countries [26,30,32]. The different variability in trends of mental disorders among the current sample can be explained by various factors. First, refugees have different characteristics and resilience capacities that make individual differences in improving or worsening mental health symptoms [63,64]. Second, since post-migration living conditions of traumatized refugees have a causal role in mental health trajectories [65], the living conditions seem to differ between the individuals in the current sample.

In the first measurement, 97.8% of participants reported at least one traumatic event. The high level of experienced traumatic events in the current study is consistent with previous findings among Syrian refugees in Turkey, Iraq, and Germany [5,15,23]. In the second measurement, although no war-related traumatic events were reported, 63% of participants still reported at least one non-war aversive or traumatic event over the last 18 months. It is troubling that Syrian refugees in the camps of Iraq are still vulnerable to experiencing new traumatic or critical life events, even after the flight from war zones and resettlement in the host country. This also suggests the vulnerability of this population to further mental health problems in the future. It is essential to mention that Syrian refugees in Iraqi camps live under difficult conditions. Although several agencies and NGOs provide many support services for Syrian refugees in Iraq, it is still insufficient to adequately address the need. According to a current joint press release by the World Food Programme (WFP) and UNHCR, without urgent support, Syrian refugees in Iraq will no longer have access to many basic supplies [66].

### Strengths and Limitation

The present study is characterized by several strengths, most of all the application of instruments that were validated for Middle Eastern populations and the fact that the interviews were conducted under supervision by locally trained psychologists and social workers who were fluent in the Kurdish Kurmanji and Arabic languages. However, the present study is clearly limited by the small sample size, which interferes with the generalization of the findings. In addition, the study did not assess more potential factors that play a role in maintaining mental problems at high levels, including daily stressors, which might also influence the trajectories of mental disorders over time. Finally, since the current study participants were married couples, the trajectories of mental disorders might differ among single people. This is because of the interdependence of trauma-related symptoms within couples, in which a husband is influenced not only by his trauma but also by his wife’s trauma as well, and vice versa [67]. Therefore, further follow-up studies on the mental health of traumatized people outside of the couples’ context are recommended.

## 5. Conclusions

The current study demonstrated that the prevalence rates of probable PTSD and depression symptoms remained high over time for a significant proportion of Syrian refugees in the present sample. Despite of their flight from war and conflict zones, they were still vulnerable to exposure to several types of traumatic events after their migration. These findings suggest that time alone is insufficient to resolve refugees’ mental illness without effective long-term mental health care. In addition, the results suggest that living conditions after migration may determine the trajectories of mental disorders over time. Without appropriate support and intervention, living in such camp conditions cannot provide Syrian refugees a positive quality of life that can enable them to thrive.

## Figures and Tables

**Table 1:** Socio-demographic information of the sample, N = 92.

Variables	t1			t2		
	M	SD	Range	M	SD	Range
Age (years)	38.53	7.02	25–57	40.44	1.10	27–59
Formal education (years)	4.36	3.61	0–14	-	-	-
N. children	4.82	1.65	0–8	5.05	1.51	2–8
Length of stay in camp (years)	2.83	1.23	0.8–6	4.97	1.10	2.3–7.5

**Table 2:** Rate of changes in mental health symptoms over 18 months, N = 92.

Variables	Percentage of Changes
PTSD	
Not changed	78.3%
Worsened	5.4%
Improved	16.3%
Depression	
Not changed	77.2%
Worsened	8.7%
Improved	14.1%

**Table 3:** Hierarchical regression analysis for variables predicting PTSD and depression change scores (t2 − t1), N = 92.

Predictors	PTSD Change Score			Depression Change Score		
	β	sr^2^	*t*	β	sr^2^	*t*
Model 1						
Gender	−0.14	−0.14	−0.128	0.18	0.17	1.70
Model 2						
Gender	−0.13	−0.11	−1.20	0.20	0.18	1.84
Age	−0.11	−0.09	−1.02	−0.05	−0.04	−0.43
N. Traumatic events—baseline	−0.05	−0.05	−0.52	0.01	0.01	0.06
N. Non-war traumatic events in the last 18 months	0.42 ***	0.41 ***	4.29 ***	0.36 ***	0.35 ***	3.66 ***

Note: *** *p* < 0.001.

**Table 4:** Correlation of significant non-war traumatic events with PTSD and depression change scores.

Traumatic Life Events over 18 Months	Frequency (%)	PTSD Change Score	Depression Change Score
Experiencing the life-threatening illnesses of a loved one.	14 (15.2%)	0.42 **	0.30 **
Experiencing a life-threatening illness or injury.	8 (8.7%)	0.32 **	27 *
Witnessing a severe transportation accident.	16 (17.4%)	0.39 **	0.22 *
Experiencing a life-threatening fire or explosion	2 (2.2%)	0.25 *	0.22 *
Experiencing a natural disaster	55 (59.8%)	0.22 *	-

Note: * *p* ≤ 0.05, ** *p* < 0.01

## Data Availability

The data will be provided by the corresponding author upon reasonable request.
